# Activation of Human CD11b^+^ B1 B-Cells by *Trypanosoma cruzi*-Derived Proteins Is Associated With Protective Immune Response in Human Chagas Disease

**DOI:** 10.3389/fimmu.2018.03015

**Published:** 2019-01-04

**Authors:** Livia Silva Araújo Passos, Luísa Mourão Dias Magalhães, Rodrigo Pinto Soares, Alexandre F. Marques, Marina Luiza Rodrigues Alves, Rodolfo Cordeiro Giunchetti, Maria do Carmo Pereira Nunes, Kenneth J. Gollob, Walderez Ornelas Dutra

**Affiliations:** ^1^Laboratory of Cell-Cell Interactions, Instituto de Ciências Biológicas, Departamento de Morfologia, Belo Horizonte, Brazil; ^2^Pós-graduação em Parasitologia, Universidade Federal de Minas Gerais, Belo Horizonte, Brazil; ^3^Laboratory of Cellular and Molecular Parasitology, Instituto René Rachou, Fundação Oswaldo Cruz, FIOCRUZ, Belo Horizonte, Brazil; ^4^Departamento de Clínica Médica, Faculdade de Medicina, Universidade Federal de Minas Gerais, Belo Horizonte, Brazil; ^5^Center for International Research, A.C.Camargo Cancer Center, São Paulo, Brazil; ^6^Instituto Nacional de Ciência e Tecnologia Doenças Tropicais, Belo Horizonte, Brazil

**Keywords:** B1 B-cells, Chagas disease, cardiomyopathy, *Trypanosoma-cruzi*, immunoregulation, cytokines

## Abstract

B-cells mediate humoral adaptive immune response via the production of antibodies and cytokines, and by inducing T-cell activation. These functions can be attributed to distinct B-cell subpopulations. Infection with *Trypanosoma cruzi*, the causative agent of Chagas disease, induces a polyclonal B-cell activation and lytic antibody production, critical for controlling parasitemia. Individuals within the chronic phase of Chagas disease may remain in an asymptomatic form (indeterminate), or develop severe cardiomyopathy (cardiac form) that can lead to death. Currently, there is no effective vaccine to prevent Chagas disease, and no treatment to halt the development of the cardiomyopathy once it is installed. The pathology associated with cardiac Chagas disease is a result of an inflammatory reaction. Thus, discovering characteristics of the host's immune response that favor the maintenance of favorable heart function may unveil important immunotherapeutic targets. Given the importance of B cells in antibody production and parasite control, we investigated *T. cruzi-*derived antigenic fractions responsible for B-cell activation and whether frequencies and functional characteristics of B-cell subpopulations are associated with different clinical outcomes of human Chagas disease. We stimulated cells from indeterminate (I) and cardiac (C) Chagas patients, as well as non-infected individuals (NI), with *T. cruzi*-derived protein- (PRO), glycolipid- (GCL) and lipid (LIP)-enriched fractions and determined functional characteristics of B-cell subpopulations. Our results showed that the frequency of B-cells was similar amongst groups. PRO, but not GCL nor LIP, led to an increased frequency of B1 B-cells in I, but not C nor NI. Although stimulation with PRO induced higher TNF expression by B1 B-cells from C and I, as compared to NI, it induced expression of IL-10 in cells from I, but not C. Stimulation with PRO induced an increased frequency of the CD11b^+^ B1 B-cell subpopulation, which was associated with better cardiac function. Chagas patients displayed increased IgM production, and activation of gamma-delta T-cells, which have been associated with B1 B-cell function. Our data showed that PRO activates CD11b^+^ B1 B-cells, and that this activation is associated with a beneficial clinical status. These findings may have implications in designing new strategies focusing on B-cell activation to prevent Chagas disease cardiomyopathy.

## Introduction

B lymphocytes are instrumental for the establishment of adaptive immune response as they mediate humoral immunity, characterized by the production of antibodies that can recognize and neutralize specific antigens. They can be classified into two major groups, B1 and B2 cells, according to their location, migration capacity and activation mechanisms (1, 2). Most studies concerning the different B cell subpopulations have been performed in experimental models, but the existence of human counterparts for these distinct B cell subpopulations has been shown (3, 4). Murine B1 B-cells are present at low frequency (0.3–0.5%) in peripheral blood, display a polyspecific B cell receptor (BCR), secrete natural antibodies spontaneously, and are activated in a T-independent fashion (5). In addition, they are self-renewing and proliferate for generations (1, 5). B2 B-cells correspond to the vast majority of B-cells and, although they can produce poly-reactive IgM antibodies (6), they are related to the production of long-lasting high affinity IgG antibodies (7). Human B-cells can also be subdivided into B1 and B2 subpopulations (3), sharing similar characteristics to the ones described for murine cells. Concomitant expression of CD19, CD20, CD27, and CD43 allow for the identification of B1, B2 memory, and B2 naïve human B-cells (8, 9). Furthermore, expression of CD11b distinguishes B1 B-cells into subpopulations associated with antibody production (CD11b^−^ B1 B-cells) and T-cell activation (CD11b^+^ B1 B-cells) (10). Different antigenic components such as soluble peptides, carbohydrates and lipid antigens can lead to the activation of the distinct B cell subpopulations (11–13).

*Trypanosoma cruzi* infection, which causes Chagas disease, a disease that leads to a potentially deadly cardiomyopathy in about 30% of the infected patients, induces a polyclonal B cell activation characterized by the production of conventional and lytic antibodies, critical for disease diagnostics and parasite control, respectively (14, 15). Despite the importance of humoral response and cytokine production by B-cells, few studies have addressed their role in human Chagas disease. It has been shown that Chagas patients have a high frequency of circulating CD5^+^ B lymphocytes (16), which, unlike activated T cells, remain at high frequencies even after parasitological cure of patients (17). Moreover, stimulation of peripheral blood mononuclear cells from Chagas patients with host-purified anti-trypomastigote antibodies leads to the expansion of CD5^+^ B-cells (18). Conventional B-cells produce regulatory cytokines and, therefore, have an immunomodulatory potential in Chagas disease (19). Other studies have shown that B-cells can also influence T cell activation either by presenting antigens or by producing activating cytokines (20). This crosstalk between B-T cells is, as of yet, unknown in Chagas disease although it may play a role in the development of protective or pathogenic immune responses.

We hypothesize that distinct B cell subpopulations may be associated with the different clinical outcomes of human Chagas disease, and may respond differently to parasite-derived components. To test this hypothesis, we sought to investigate which *T. cruzi*-derived fractions are responsible for the activation of different B cell subpopulations, and whether their frequencies and functional characteristics are associated with the distinct clinical status of patients with Chagas disease. Our results showed that a *T. cruzi*-derived protein-enrich fraction (PRO), but not glycolipid- (CGL) or lipid-enriched (LIP) fractions, led to an increase in the frequency of B1 B-cells in indeterminate, but not cardiac Chagas patients, and that these cells were associated with better cardiac function. Activated CD11b^+^ B1 B-cells from indeterminate patients express TNF but also the anti-inflammatory cytokine IL-10 upon stimulation with PRO, suggesting that B1 B-cells from indeterminate patients display balanced immune response. On the other hand, PRO induces an inflammatory profile in B-cells from cardiac patients. In addition, indeterminate patients display a higher frequency of activated CD4^+^gamma-delta^+^ T cells and CD4^−^CD8^−^ gamma-delta^+^ T cells *ex vivo* and after PRO stimulation, as compared to non-infected individuals and cardiac patients. These findings identify a protein-enriched fraction as the parasite component that stimulate B1 B-cells from indeterminate Chagas patients. Given the association with a protective response and better cardiac function, these findings may have implications in designing strategies for prevention of Chagas disease cardiomyopathy, and other cardiomyopathies where B1 B-cell activation may play an important role.

## Methods

### Patients

Patients with well-defined clinical forms of Chagas disease, as well as non-Chagas individuals were enrolled in this cross-sectional study, which has the approval of the Ethical Committee from Federal University of Minas Gerais (COEP-UFMG–ETIC006/05), and is in accordance with the Declaration for Helsinki. Treatment and clinical care was offered to all volunteers, despite their enrollment in this research project. Twelve volunteer patients were carefully selected to be unequivocally within the indeterminate and dilated cardiac clinical forms of Chagas disease. Standard serology exams for Chagas disease, electrocardiogram and echocardiogram, physical examinations and chest X-rays were performed with the purpose of characterizing the clinical status of the patients, as previously defined (21). Patients from asymptomatic clinical form (Indeterminate Chagas patients – I; 4 females, 2 males) had positive serology, lack of clinical manifestations or alterations upon all clinical, radiological and echocardiographic examination. Cardiac Chagas patients (C; 3 females, 3 males) displayed positive serology, right and/or left ventricular dilation, global left ventricular dysfunction, alterations in the cardiac electric impulse generation and conduction upon electrocardiogram, chest x-rays, and echocardiography. Measures of left ventricular ejection fraction (LVEF) and left ventricular diastolic diameter (LVDD) were used as parameters to express disease severity (21). The normal range of LVDD and LVEF are 42–58 mm and 52–72%, respectively. Patients were from Chagas disease endemic areas within Minas Gerais, Brazil, and have been evaluated at the outpatient clinic of the Universidade Federal de Minas Gerais. Six individuals who displayed negative specific serological tests for Chagas disease, from the same geographical region, were included as non-infected group (NI; 3 females, 3 males). Any other chronic inflammatory diseases, diabetes, heart/circulatory illnesses or bacterial infections were used as exclusion criteria. The average age of the patients did not statistically differ amongst groups. Table 1 summarizes the clinical characteristics of the individuals enrolled in this study.

**Table 1:** Non infected individuals and patients with Chagas disease analyzed in the study.

**ID**	**Clinical form**	**Age range per group (years)**	LVDD(mm)^*^	LVEF(%)^*^
NI1	Non-infected		ND	ND
NI2	Non-infected		ND	ND
NI3	Non-infected		ND	ND
NI4	Non-infected		ND	ND
NI5	Non-infected		ND	ND
NI6	Non-infected	24–57	ND	ND
I1	Indeterminate		50	71
I2	Indeterminate		48	60
I3	Indeterminate		41	64
I4	Indeterminate		50	55
I5	Indeterminate		53	60
I6	Indeterminate	18–72	48	68
C1	Cardiac		75	21
C2	Cardiac		75	25
C3	Cardiac		48	62
C4	Cardiac		67	26
C5	Cardiac		48	66
C6	Cardiac	51–63	48	66

NI, non-infected individuals; I, indeterminate patients; C, cardiac patients; ID, identifier; LVDD, left ventricular diastolic diameter; LVEF, left ventricular ejection fraction, ND, not determined.

* *p < 0.05, when comparing the values of LVDD and LVEF between indeterminate and cardiac patients*.

### Parasites and Antigen Fractions

CL Brener trypomastigotes of *T. cruzi* were grown in VERO cells, as previously performed by us (22) until obtaining a total of 2 × 10^9^ parasites. In short, cells were infected with ten trypomastigotes/cell and non-internalized trypomastigotes were removed by washing with RPMI culture media (Sigma-Aldrich, St. Louis, US) supplemented with 5% inactivated fetal calf serum and antibiotic -penicillin 500 U/mL and streptomycin 0,5 mg/mL, followed by incubation for approximately 6 days. After this period, trypomastigotes ruptured the cells, and were collected from the supernatant, centrifuged and then washed twice with PBS (Sigma-Aldrich, St. Louis, US) by centrifugation (800 g for 5 min at 4°C). Parasites obtained in such a manner were stored at −80°C as dry pellet, used to prepare fractions.

Antigenic fractions of *T. cruzi* were obtained using the methodology proposed by Gazos-Lopes et al. (23) with adaptations (24). Frozen pellets obtained as described above were suspended in 1.6 mL of ultrapure water (W) and transferred to 13 × 100 mm polytetrafluoroethylene (PTFE)-lined screw cap Pyrex culture tubes. Chloroform (Ch) and methanol (M) (1:2 v/v) were added to each vial and samples were mixed vigorously using a vortex for 2 min and then centrifuged for 15 min at 1,800 g at room temperature; this process was repeated three times. Pellet was extracted once again with Ch/M (2:1, v/v), samples were mixed vigorously for 2 min using a vortex and then centrifuged for 15 min at 1,800 g at room temperature. At the end of each extraction step, insoluble trypomastigote components were stored for later extraction of proteins. Supernatants were transferred to (PTFE)-lined Pyrex glass test tubes and then dried on a lyophilizer. Next, samples were pooled together and subjected to Folch's partition (25). For this, samples were dissolved in Ch/M/W (4:2:1.5, v/v/v) and then mixed vigorously for 5 min using a vortex, and finally centrifuged for 15 min at 1,800 g at room temperature. After centrifugation, a lower (organic) phase, containing lipids, and an upper (aqueous) phase containing glycolipids were obtained. Once again, samples were dried by lyophilization. The lipid-enriched fraction (LIP) was resuspended in 1,0 mL of dimethyl sulfoxide (DMSO—Sigma-Aldrich, St. Louis, US), and 1,0 mL of ultrapure water was added to the tube containing the glycolipid-enriched fraction (GCL).

For *T. cruzi* protein extraction, we used the parasite components remaining from LIP and GCL extraction, as described Almeida et al. (26). In brief, insoluble material was extracted three times using 10 volumes of butan−1–ol-saturated water (9% butan−1–ol) under agitation at room temperature for 4 h. The resulting extracts were collected in a single sample and dried on the lyophilizer. Protein-enriched fraction (PRO) was resuspended in 1,0 mL of ultrapure water. All fractions were in stored in sterile glass tubes at −80°C until use.

### Blood Sampling and *in vitro* Cultures

Peripheral blood samples were collected in heparin tubes through venipuncture from all eighteen volunteers enrolled in this study. Peripheral blood mononuclear cells (PBMCs) were obtained by differential centrifugation using Ficoll (Sigma-Aldrich, St. Louis, US), as routinely done by us (22). The cells were washed and resuspended in RPMI 1640 medium(Sigma-Aldrich, St. Louis, US) supplemented with 5% heat-inactivated AB human serum (Sigma-Aldrich, St. Louis, US), antibiotics (penicillin, 200 U/mL; and streptomycin, 0.1 mg/mL) and L-glutamine (1 mM) (Sigma-Aldrich, St. Louis, US) at a concentration of 1 × 10^7^ cells/mL. Cells from each volunteer were placed on 96 well plates (Corning®, Corning, US) in 200 μL cultures under the following conditions: medium alone, GCL antigen (20 μg/mL), LIP antigen (equal to 5 parasites) and PRO antigen (equal to 10 parasites) for 18 and 36 h, depending on the measurement, as indicated in figure legends. GCL antigen concentration was measured by phenol–sulfuric acid method (27), while LIP and PRO antigens were extrapolated from the number of parasites used for the extraction. Brefeldin A (1 μg/mL) was added for last 4 h of culture to prevent cytokine secretion.

### Immunophenotyping

Immunophenotypic analyses of PBMCs exposed to the different stimuli were performed by multiparametric digital flow cytometry, to determine the activation status and cytokine production by the B cell subpopulations. Stimulated (treated as described above) or non-stimulated cells were harvested after the final 18 h of culture and submitted to specific staining. We used combinations of monoclonal antibodies (mAbs) specific for human leukocytes cell-surface markers, including: BV510 labeled anti-CD19 (clone HIB19), BV421 labeled anti-CD20 (clone 2H7), APCCy7 labeled anti-CD27 (clone O323), APC labeled anti-CD43 (clone 10G7), FITC labeled anti-CD11b (clone 6B11) to identify the specific subpopulations of B-cells and BV510 labeled anti-CD4 (clone OKT4), PECy5.5 labeled anti-CD8 (clone OKT8), BV421-labeled anti-TCRαβ (clone IP26), and FITC-labeled anti-TCRγδ (clone B1). 40μl mixture of these surface marker-specific antibodies were added to each well of a 96-round bottom plate (Corning®, Corning, US) containing 3 × 10^5^ cells, for 15 min at 4°C. After incubation, samples were washed in phosphate-buffered saline (PBS- (Sigma-Aldrich, St. Louis, US) containing 1% bovine serum albumin (BSA—Sigma-Aldrich, St. Louis, US), and fixed by 20 min incubation with a 2% formaldehyde solution. After removal of the fixing solution by centrifugation, and washing with PBS, we permeabilized the cells by incubation for 15 min with a 0.5% saponin solution, and proceeded to the intracellular staining. Samples were incubated with PECy7 labeled anti-IL-10 (clone JES3-9D7), PE labeled anti-TNF (clone MEM-56) monoclonal antibodies for 20 min at room temperature, washed twice with 0,5% saponin solution, resuspended in PBS and read in a flow cytometer. All antibodies were from BioLegend (San Diego, US). A minimum of 150,000 gated events from each sample were collected using a FACS Canto II (Becton Dickinson—San Jose, US), and analyzed using the FlowJo software (San Jose, US).

### Measurement of IgM and IgG Antibodies From Supernatants of Cultures Stimulated With *T. cruzi*-Derived PRO-Enriched Fractions

IgM and IgG immunoglobulins were measured in the supernatants of cultures of cells from non-infected individuals (NI) and Chagas disease patients (CD) stimulated with *T. cruzi-*derived PRO-fraction. To do so, we use the commercial kits (Total Ready-SET-Go Human IgM! and Total Human IgG Ready-SET-Go!, eBioscience, CA, US) and the tests were performed according to the manufacturer's instructions. Briefly, 96 wells flat bottom plates were sensitized overnight at 4°C, washed twice using the wash buffer solution provided by the manufacturer. Subsequently, blocking buffer was added to all wells and the plate and was incubated during 2 h at room temperature. After 2 h of incubation, the plates were again washed three times with wash buffer. Aliquots from supernatants of cultures (diluted 1:2 for both IgM and IgG) were added to the wells of the plates and incubated for 2 h under agitation at room temperature. Plates were washed with wash buffer 4 times and then the detection antibodies were added to the all wells for a period of 2 h under agitation at room temperature. Three more washing steps were performed and the solution containing the substrate was added to the wells for 15 min at room temperature. Finally, the reaction was stopped and the absorbance values of each of the wells were acquired in an ELISA reader at 450 nm. Absorbance values were converted to concentration values (ng/mL) using the standard curve. Standard curve was made by serial dilution using reagents provided in the manufacturer.

### Statistical Analysis

All data showed a Gaussian distribution, as determined by Kolmogorov-Smirnov test. Paired *T*-test was used to ascertain differences between unstimulated cultures and stimulated cultures within the same group of patients. Correlation analyzes were performed using Pearson's coefficient. Comparisons between different groups were performed using unpaired *T*-test. Differences that returned *p* ≤ 0.05 were considered statistically significant from one another.

## Results

### Stimulation With *T. cruzi*-Derived Protein Fraction Leads to Higher Frequency of B1 B-Cells in Cell Cultures From Indeterminate but Not Cardiac Chagas Patients

**Figure 1:**
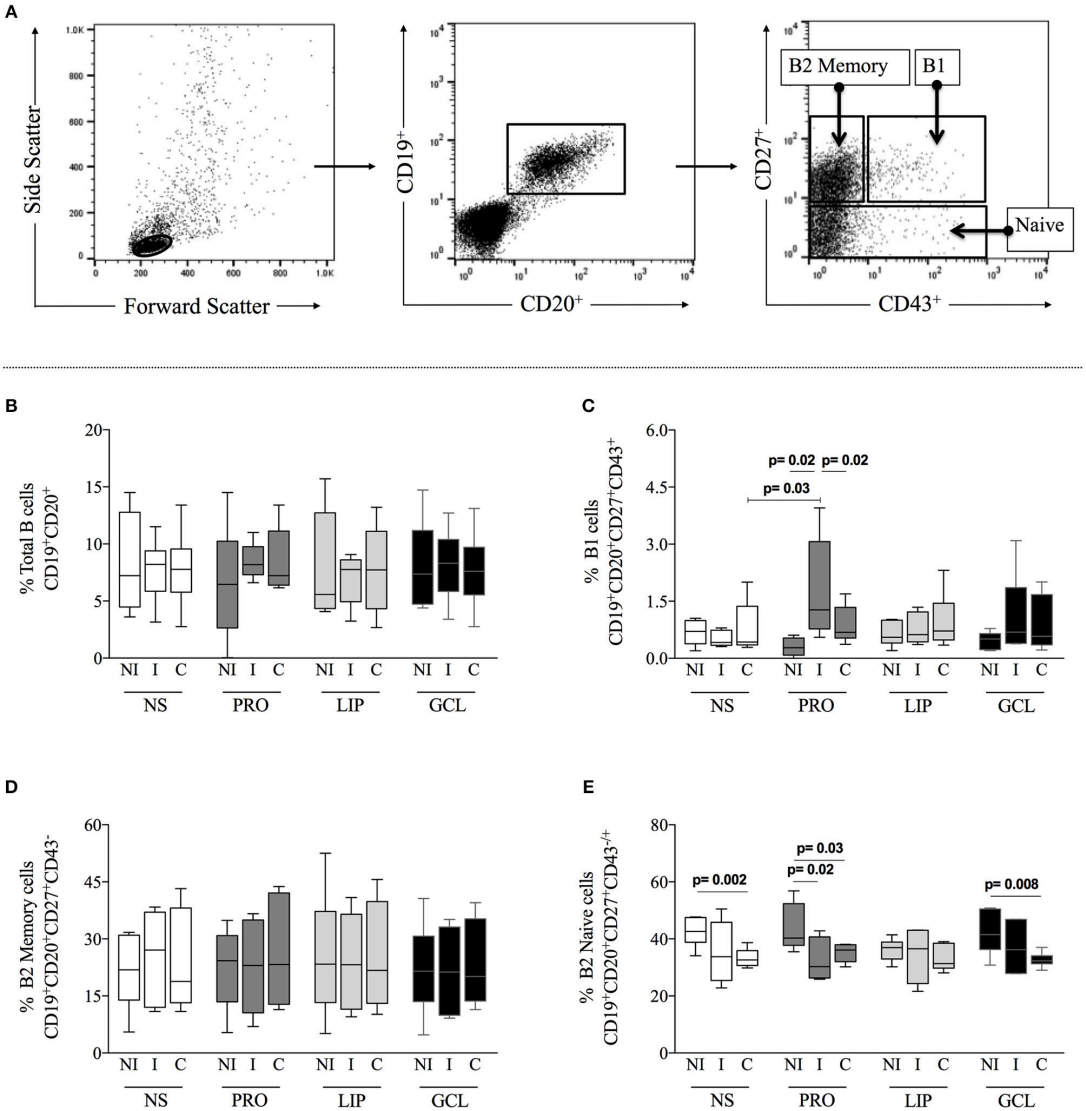
Analysis of total B-cells and subpopulations. **(A)** Representative dot plots illustrating the gating strategy to access total B-cells (CD19^+^CD20^+^) within gated lymphocytes from total peripheral blood mononuclear cells (PBMCs), and B-cells subpopulations, B1 (CD27^+^CD43^+^), B2 Memory (CD27^+^CD43^−^) and B2 Naïve (CD27^−^CD43^−^/^+^) from total B-cells. PBMCs were obtained from non-infected individual (NI), indeterminate Chagas patients (I) and cardiac Chagas patients **(C)** and analyzed in non-stimulated cultures (NS) – white box plot, or stimulated with protein enriched fraction (PRO)- dark gray box plot, or lipid enriched fraction (LIP) - gray box plot or glycolipid enriched fraction (GCL) black box plot. **(B)** Analysis of the frequency of total B-cells. **(C)** Analysis of the frequency of total B1 cells. **(D)** Analysis of the frequency of total B2 Memory cells. **(E)** Analysis of the frequency of total B2 Naive cells. The results are expressed as percentage. The box extends from the 25th percentile to 75th percentile, with a horizontal line at the median (50th percentile). Whiskers extend from the lowest value to the 25th percentile and from the 75th percentile to the highest value, showing the range of data distribution. Statistical significance is indicated in each graph.

First, we evaluated the frequency of total B-cells (CD19^+^CD20^+^), B1 cells (CD19^+^CD20^+^CD27^+^CD43^+^), B2 memory (CD19^+^CD20^+^CD27^+^CD43^−^), and B2 B naïve (CD19^+^CD20^+^CD27^−^CD43^±^) cells from indeterminate (I) and cardiac Chagas (C) patients, as well as from non-infected individuals (NI) under different culture conditions, as described above. Figure 1A shows the gating strategy used in the analysis. We observed that the frequencies of total B-cells, B1 cells, and B2 memory did not change significantly when comparing non-stimulated cultures (NS) amongst groups (Figures 1B–D), while the frequency of B2 naïve cells was lower in C, as compared to NI (Figure 1E). We then asked whether stimulation with parasite-derived proteins (PRO), lipids (LIP), or glycolipid (GCL)-enriched fractions induced changes in the frequency of the different B cell subpopulations. We observed that although the frequency of total B-cells and B2 memory cells was not significantly altered after stimulation with the different components (Figures 1B,D), stimulation with PRO lead to a significant increase in the frequency of B1 B-cells from I patients, as compared to NI or C patients submitted to the same stimulus, as well as compared to NS cells (Figure 1C). There was a lower frequency of B2 B naïve cells in cultures of cells from I and C patients after stimulation with PRO, as compared to NI group submitted to the same culture condition (Figure 1E). Similarly, a lower frequency of B2 B naïve cells was observed in cultures of cells from C patients stimulated with GCL as compared to cells from NI individuals submitted to the same culture conditions, but these frequencies were not different to the comparisons made amongst the groups with NS cultures (Figure 1E). Thus, stimulation with PRO leads to a higher frequency of B1 B cells in cultures from indeterminate Chagas patients.

### B1 B-Cells From Indeterminate Patients Produce TNF-α but Also IL-10 Before and After Stimulation With *T. cruzi*-Derived Protein Fraction

**Figure 2:**
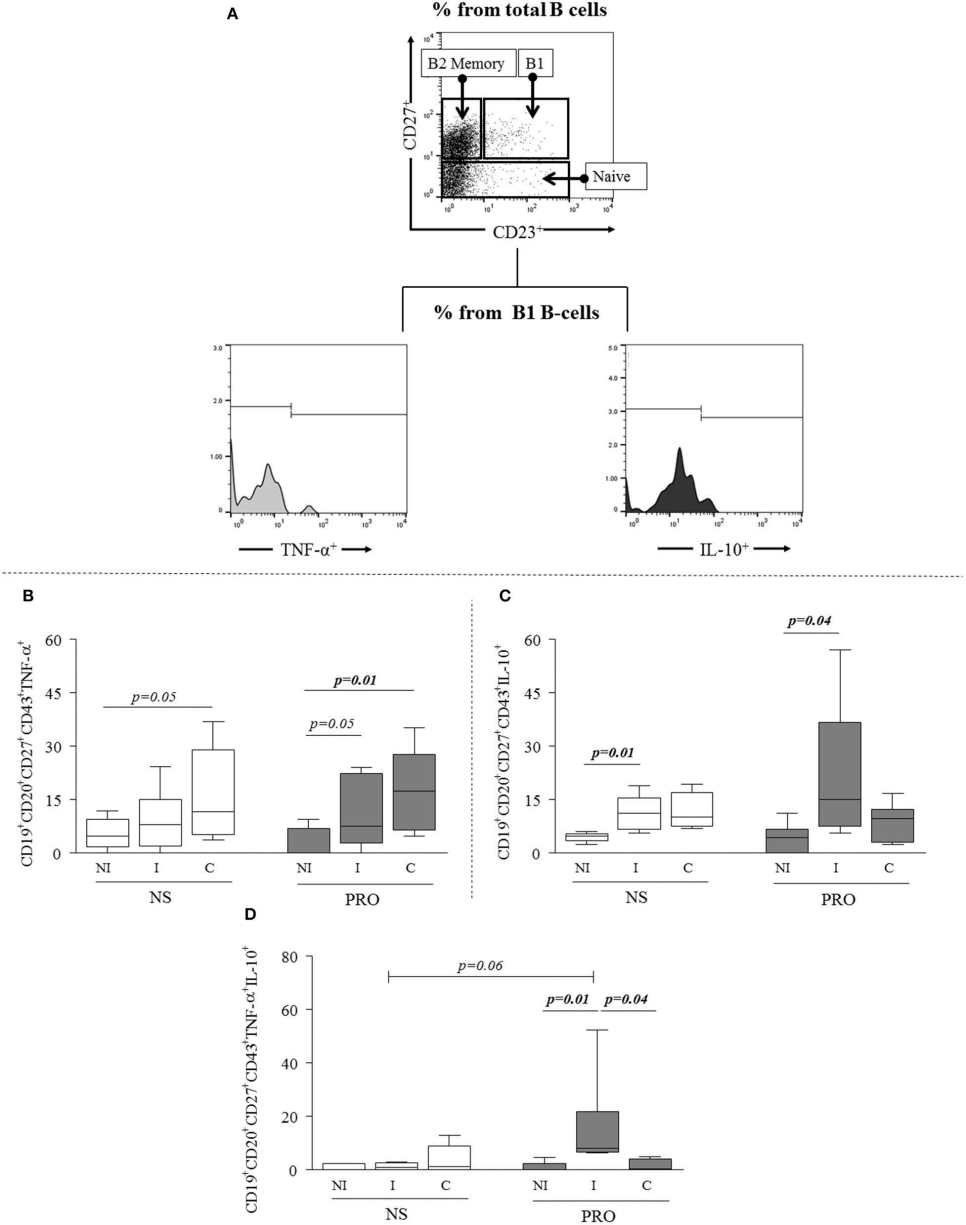
Analysis of cytokine expression by B1 B-cells. **(A)** Representative dot plots and histogram illustrating the gating strategy to access frequency of expression of cytokines TNF-α and IL-10 in B1 B-cells subpopulations. **(B)** Expression of TNF-α by B1 B-cells (CD19^+^CD20^+^CD27^+^CD43^+^TNF-α^+^) in non-stimulated cultures –and PRO stimulated cultures. **(C)** Expression of IL-10 by B1 B-cells (CD19^+^CD20^+^CD27^+^CD43^+^IL-10^+^) in non-stimulated cultures and PRO stimulated cultures. **(D)** Concomitant expression of TNF-α and IL-10 (CD19^+^CD20^+^CD27^+^CD43^+^TNF-α^+^ IL-10^+^) by B1-cells in non-stimulated cultures and PRO stimulated cultures. White box blot represents non-stimulated cultures and dark gray box plot represents PRO stimulated cultures from non-infected individuals (NI), indeterminate Chagas patients (I) and cardiac Chagas patients **(C)**. The results are expressed as percentages. The box extends from the 25th percentile to 75th percentile, with a horizontal line at the median (50th percentile). Whiskers extend from the lowest value to the 25th percentile and from the 75th percentile to the highest value, showing the range of data distribution. Statistical significance is indicated in each graph.

Next, we sought to determine if the PRO-stimulated B1 B-cells were committed to the expression of cytokines and antibodies. We evaluated the expression of inflammatory and anti-inflammatory cytokines, represented by TNF-α and IL-10, respectively (Figure 2A). We observed that the frequency of TNF-α- producing B1 B-cells from C but not I patients was slightly higher than the one observed in NI individuals without any stimulation (*p* = 0.05), and that PRO stimulation significantly increased the frequency of TNF-α producing B1 B-cells in cultures of cells from C patients, and slightly increased the expression of this cytokine by B1 B-cells from I (*p* = 0.05; Figure 2B). Unstimulated B1 B-cells from I displayed higher expression of IL-10 than B1 B-cells from NI individuals (Figure 2C). PRO-stimulation further increased the frequency of IL-10^+^ B1 B-cells from I patients as compared to NI individuals submitted to the same stimulation (Figure 2C). Moreover, when we evaluated co-expression of these cytokines by B1 B-cells before and after PRO stimulation we found that the frequency of double positive cells (TNF-α^+^IL−10^+^) was greater in I patients as compared to NS and C in PRO-stimulated cultures (Figure 2D).

**Figure 3:**
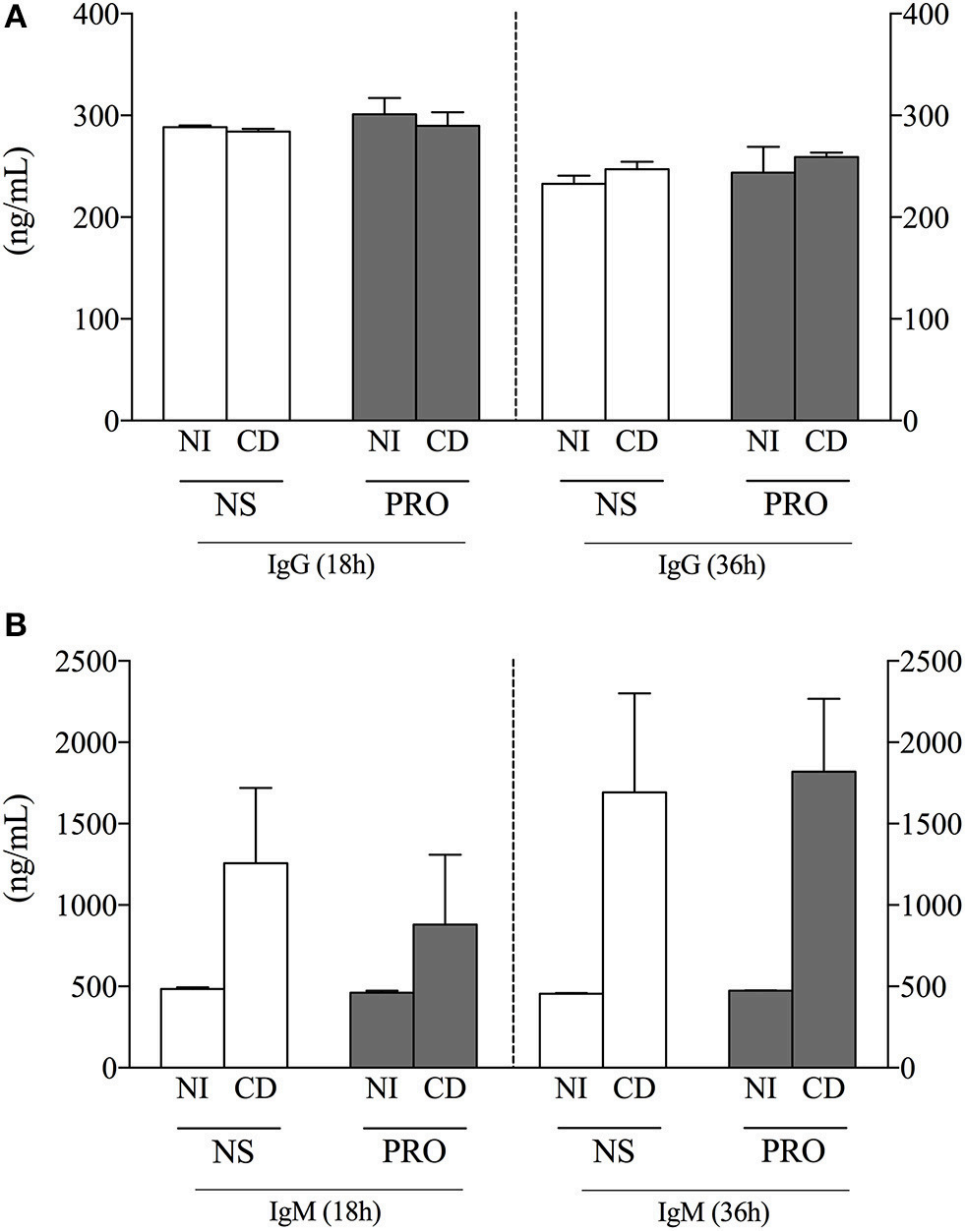
Analysis of IgG and IgM production. **(A)** IgG concentration (ng/mL) in cell culture supernatant from non-infected individuals (NI) and Chagas patients (CD) in non-stimulated cultures (NS) and PRO stimulated cultures. **(B)** IgM concentration (ng/mL) in cell culture supernatant from non-infected individuals (NI) and Chagas patients (CD) in non-stimulated cultures (NS) and PRO stimulated cultures. The results are expressed as percentage in bar graphs. The bars represent the mean of individual values with standard error of the mean.

In order to determine another important functional characteristic of B-cells, we measured antibody secretion in supernatants of cultures from NI individuals and Chagas disease patients (CD) stimulated or not with PRO fraction. We observed that the secretion of IgG did not vary comparing between stimulated and non-stimulated cultures or between CD and NI individuals (Figure 3A). Interestingly, while IgM secretion was low in cultures from NI individuals before and after stimulation, secretion of IgM was high in cultures from CD in unstimulated as well as PRO-stimulated cultures (Figure 3B). The secretion of IgM was also higher after 36 h of culture, suggesting the continuous activation of IgM-producing cells in Chagas patients (Figure 3B). Of note, the secretion of IgM was much higher than the secretion of IgG in stimulated and non-stimulated cultures from NI as well as CD.

These results show that B1 B-cells from indeterminate and cardiac patients display distinct cytokine expression in response to PRO, associated with predominant anti-inflammatory and inflammatory responses, respectively, and that PRO sustains high IgM production by B cells from Chagas patients.

### Expansion of Activated CD11b^+^ B1 B-Cells Upon Stimulation With *T. cruzi*-Derived Proteins Is Associated With Better Cardiac Function

**Figure 4:**
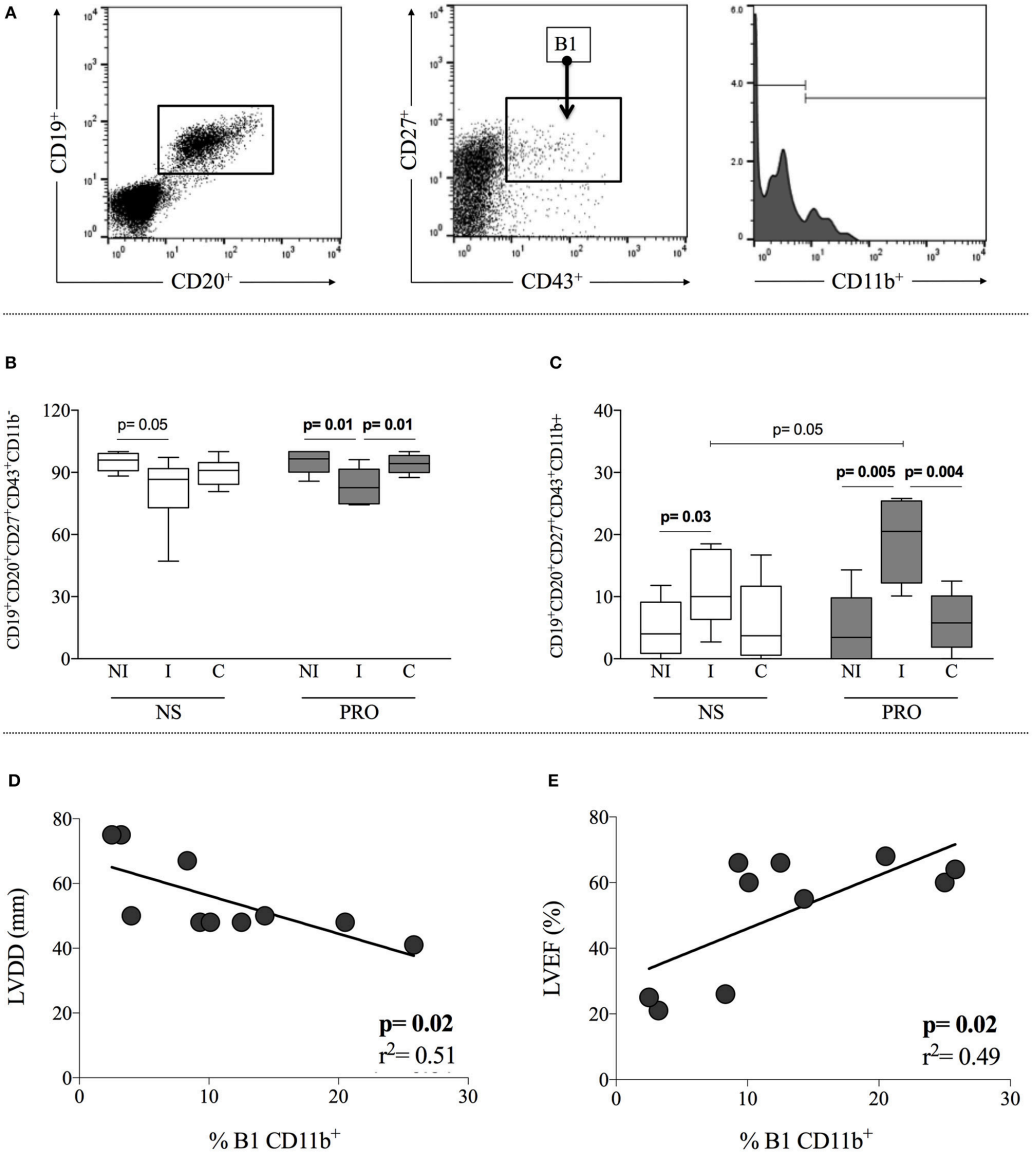
Analysis of CD11b expression in B1 B-cells and correlation with clinical parameters. **(A)** Representative dot plot and histogram illustrating the gating strategy to access frequency of expression CD11b^+^ and CD11b^−^ in B1 B-cells subpopulation **(B)** Frequency of CD11b^+^ B1 B-cells (CD19^+^CD20^+^CD27^+^CD43^+^CD11b^+^) in non-stimulated cultures—white box plot and PRO stimulated cultures—dark gray box plot from non-infected individuals (NI), indeterminate Chagas patients (I) and cardiac Chagas patients **(C)**. **(C)** Frequency of CD11b^−^ B1 B-cells (CD19^+^CD20^+^CD27^+^CD43^+^CD11b^−^) in non-stimulated cultures - white box plot and PRO stimulated cultures—dark gray box plot from non-infected individuals (NI), indeterminate Chagas patients (I) and cardiac Chagas patients **(C)**. The results are expressed as percentage. The box extends from the 25th percentile to 75th percentile, with a horizontal line at the median (50th percentile). Whiskers extend from the lowest value to the 25th percentile and from the 75th percentile to the highest value, showing the range of data distribution. **(D,E)** Correlation analysis showing a negative and positive correlation between B1 CD11b^+^ and left ventricular diastolic diameter (LVDD) left ventricular ejection fraction (LVEF), respectively. Statistical significance is indicated in each graph.

Expression of CD11b is associated with activation of B1 B-cells and defines a sub-population that displays T-cell activation properties (10). Thus, we further subdivided the B1 B-cells according to CD11b expression, discriminating CD11b^+^ and CD11b^−^ subpopulations. Gating strategy is shown in Figure 4A. We observed that I patients display a higher frequency of CD11b^+^ B1 B-cells as compared to NI individuals, in cultures without any stimulation (Figure 4B). This frequency was further amplified in cultures of cells from I patients by stimulation with PRO (Figure 4B), where the frequency of CD11b^+^ B1 B-cells was higher than the ones observed in NI and C after PRO stimulation, as well as higher than I before stimulation. This expansion was not observed in cultures of cells from C patients (Figure 4A). The increased frequency of CD11b^+^ B1 B-cells in cultures of cells from I patients was compensated by a decrease in CD11b^−^ B1 B-cells (Figure 4C).

Given the association of the frequency of B1 B-cells with the I form of Chagas disease, we asked whether there was a correlation between the frequency of these cells and clinical parameters of cardiac function. In order to address this point, we performed correlative analysis between the frequency of CD11b^+^ B1 B-cells and left ventricular diastolic diameter (LVDD), as well as left ventricular ejection fraction (LVEF). These parameters have been associated with cardiac function in Chagas disease (21), where the lower the LVDD and the higher the LVEF, the better the cardiac function. Our analysis demonstrated a negative correlation between the frequency of PRO-stimulated CD11b^+^ B1 B-cells and LVDD and a positive correlation with LVEF (Figures 4D,E, respectively), suggesting that the higher the frequency of activated B1 B-cells, the better the cardiac function of Chagas patients. Our data shows that PRO induces a higher frequency of CD11b^+^ B1 B cells in cultures of cells from indeterminate patients, and that there is a positive correlation of the frequency of this subpopulation and better cardiac function.

### PRO Stimulation Maintained the High Frequency of Activated CD4^+^ TCRgamma-Delta^+^ T Cells and Induced the Expansion of Activated CD8^+^ TCRgamma-Delta^+^ T Cells From Indeterminate Chagas Patients

**Figure 5:**
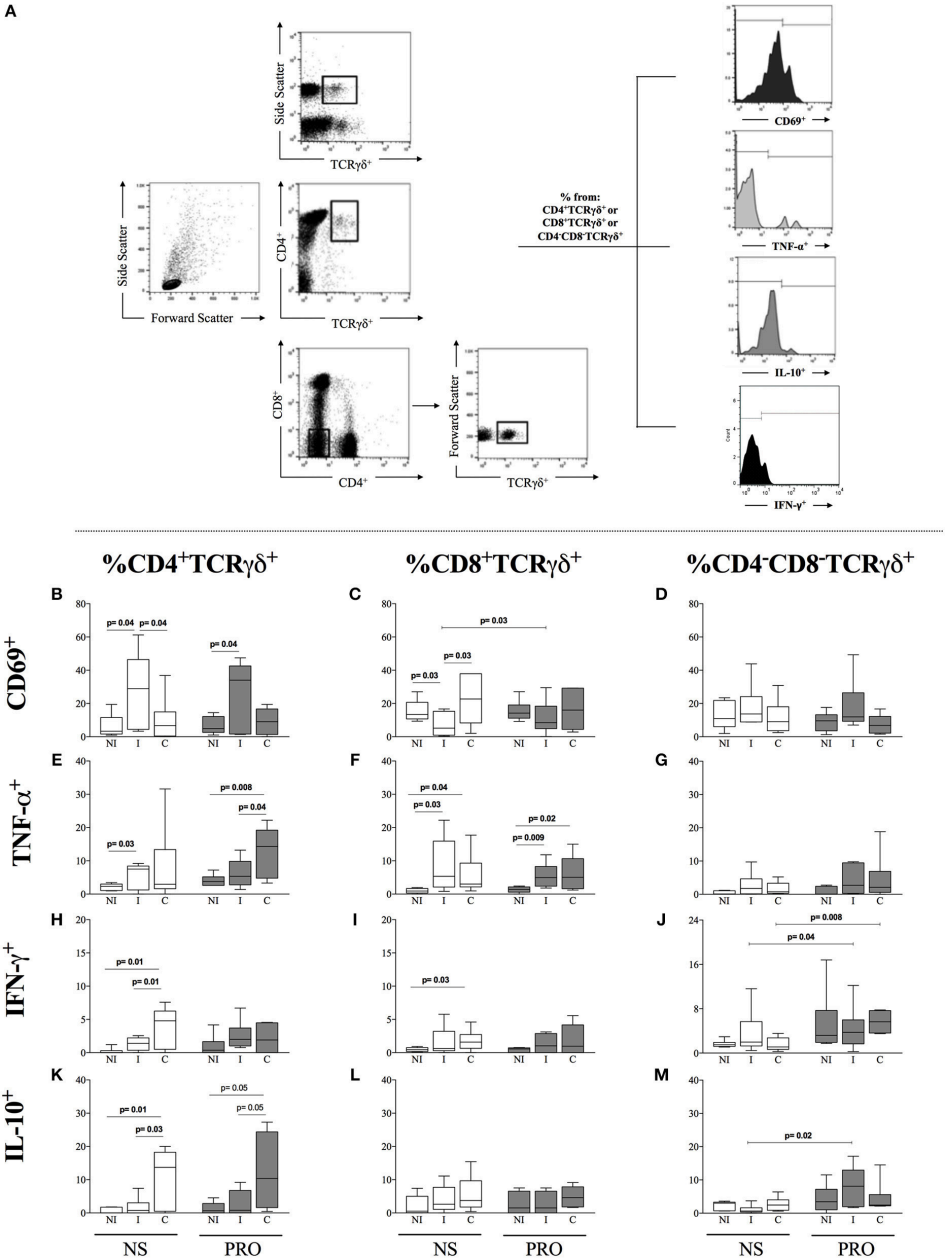
Analysis of the frequency of CD69, TNF-α, and IL-10 in CD4, CD8, and double-negative (DN/CD4^−^CD8^−^) cells expressing gamma-delta TCR. **(A)** Representative dot plots and histograms illustrating the gating strategy to access frequency of expression of cytokines TNF-α and IL-10 in TCR gamma-delta^+^ cells **(B)** Frequency of CD4^+^TCRγδ^+^CD69^+^ lymphocytes in non-stimulated cultures. **(C)** Frequency of CD8^+^TCRγδ^+^CD69^+^ lymphocytes. **(D)** Frequency of CD4^−^CD8^−^TCRγδ^+^CD69^+^ lymphocytes. **(E)** Frequency of CD4^+^TCRγδ^+^TNF-α^+^ lymphocytes. **(F)** Frequency of CD8^+^TCRγδ^+^TNF-α^+^ lymphocytes. **(G)** Frequency of CD4^−^CD8^−^TCRγδ^+^TNF-α^+^. **(H)** Frequency of CD4^+^TCRγδ^+^IFN-γ^+^. **(I)** Frequency of CD8^+^TCRγδ^+^ IFN-γ^+^. **(J)** CD4^−^CD8^−^TCRγδ^+^ IFN-γ^+^. **(K)** Frequency of CD4^+^TCRγδ^+^IL-10^+^. **(L)** Frequency of CD8^+^TCRγδ^+^IL-10^+^. **(M)** CD4^−^CD8^−^TCRγδ^+^IL-10^+^ White box plots represent non-stimulated cultures and dark gray box plots represent PRO stimulated cultures from non-infected individuals (NI), indeterminate Chagas patients (I) and cardiac Chagas patients **(C)**. **(C)** The results are expressed as percentage in box plot format. The box extends from the 25th percentile to 75th percentile, with a horizontal line at the median (50th percentile). Whiskers extend from the lowest value to the 25th percentile and from the 75th percentile to the highest value, showing the range of data distribution. Statistical significance is indicated in each graph.

Recent studies have suggested that activation of B-cells, especially activated B1 B-cells, leads to the activation of TCRgamma-delta^+^ T cells (28–30). Since we observed an increased frequency of activated B1 B-cells in cultures of cells from I patients stimulated with PRO, we determined the frequency of CD69, TNF-α, IFN-γ, and IL-10 in CD4^+^,CD8^+^, and double-negative (DN/CD4^−^CD8^−^) TCRgamma-delta^+^ T cells, the latter being the majority population of gamma delta T cells, from I and C patients, as well as NI. We observed that I patients display a higher frequency of CD4^+^CD69^+^TCRgamma-delta^+^ T cells as compared to NI and C individuals in non-stimulated cultures and that this high frequency was maintained after PRO stimulation (Figure 5B). On the other hand, I patients had a lower frequency of CD8^+^CD69^+^TCRgamma-delta^+^ T cells as compared to NI individuals and C patients in unstimulated cultures. PRO slightly increased the frequency of CD8^+^CD69^+^TCRgamma-delta^+^ cells in cultures of cells from I as compared to non-stimulated cultures, but the increased frequency was not significantly different than the frequency of these cells in PRO-stimulated cultures from NI or C (Figure 5C). Statistically significant differences in the expression of CD69 in double negative T cells were not observed (Figure 5D). We evaluated the expression of TNF-α and IL-10 by these different TCR gamma-delta^+^ T cell subpopulations. We observed that the stimulation with PRO maintained the increased expression of TNF-α by CD4^+^ TCR gamma-delta^+^ cells from C in relation to NI and I, although this increase was not statistically significant as compared to non-stimulated cultures (Figure 5E). Similar results were observed in the CD8^+^ TCR gamma-delta^+^ T cells (Figure 5F). PRO stimulation did not induce TNF-α expression by DN (CD4^−^CD8^−^) TCR gamma-delta^+^ T cells (Figure 5G). PRO did not increase the expression of IFN-γ in CD4^+^ and CD8^+^ T cells (Figures 5H,I), but increased the frequency of DN gamma-delta^+^ expressing this cytokine (Figure 5J). PRO stimulation maintained the high frequency of IL-10 expression by CD4^+^ TCR gamma-delta^+^ T cells from C, as compared to NI and I (Figure 5K). While no changes were observed in the expression of IL-10 by CD8^+^ TCR gamma-delta^+^ T cells (Figure 5L), the frequency of IL-10 expression by DN TCR gamma-delta^+^ T cells was significantly increased upon PRO stimulation of cells from I but not C nor NI (Figure 5M). Thus, PRO maintains a more balanced cytokine profile in DN gamma-delta T cells from I, as compared to C.

## Discussion

Most studies concerning B-cell responses in *T. cruzi* infection have been performed in experimental models, showing that these cells are polyclonally activated during infection, and have been associated with protective and pathogenic properties (31–34). Although B-cell responses are critical for human Chagas disease diagnosis and for parasite control (14, 15), little is known about the role of these cells in the immune response of chronic Chagas patients. In this work, we identified the *T. cruzi*-derived components responsible for the activation of human B cell subpopulations in chronic Chagas patients, and determined their contribution to the immune response observed in patients with polar clinical forms of the disease. Our data showed that a *T. cruzi*-derived protein enriched fraction (PRO) activated B1 B-cells from Chagas patients, and suggested a protective role for this particular B cell subpopulation in human Chagas disease. Supporting this hypothesis are the following findings: (1) the frequency of activated B1 B-cells from indeterminate (I), but not cardiac (C) patients, is increased upon stimulation with PRO but not LIP nor GCL; (2) these cells express the inflammatory cytokine TNF, but also the regulatory IL-10 upon stimulation with PRO, indicating a balanced response; (3) the higher frequency of activated B1 B-cells is associated with clinical parameters of better cardiac function.

The fraction obtained from trypomastigotes by extraction with 9% butanol solution, named “PRO,” contains mainly transialidases (TS) and mucin-associated surface proteins (MASPs), which are highly immunogenic glycosylphosphatidylinositol (GPI)-linked glycoproteins, and are amongst the most abundant molecules found in trypomastigote infective forms of *T. cruzi* (26, 35). Although we did not specifically identify which exact component of PRO led to the expansion of B1 B-cells, the recognition of the glycan portion of the above mentioned glycoproteins by the B-cell receptor (BCR) emerges as a possibility, since this subpopulation of B lymphocytes responds strongly to glycans in a T-independent manner (3, 36). A mechanism of T-independent B cell activation by *Borrelia hermsii* antigens and porins from *Salmonella* has previously been shown, supporting this hypothesis (37). A proline racemase identified in *T. cruzi* as an intracellular or membrane-bound protein present in infective stages of the parasite is also a possible target of this response, since this protein was shown to induce IgM, and to display B-cell mitogenic properties (38, 39). The fact that the stimulation was observed particularly in cells from I patients, and is associated with better cardiac function, may suggest a potential for protection of the stimulating component. Further studies will be performed to pinpoint the exact component (or components) from PRO that induces this potentially protective B1 B-cell-mediated response.

B1 B-cells are the main producers of natural antibodies, a group of immunoglobulins, predominantly IgM, found in individuals who have not had any previous known exposure to the antigens or immunization (40, 41). We observed that, although non-infected individuals produce IgM, Chagas patients display a high production of IgM antibodies before and after stimulation with PRO (Figure 3B). Natural IgM antibodies are produced in a constitutively and in a polyspecific manner, which is consistent with our findings. The IgM levels was higher in 36-h cultures than in 18 h (Figure 3B), suggesting that continuous exposure to *T. cruzi* antigens, as occurs in chronically infected patients, may intensify or assist in the maintenance of production of the natural antibodies. A protective role of IgM antibodies has been associated with the fact that single natural antibodies can heteroligate to different antigens thereby increasing effective avidity (42). Moreover, these antibodies forms a pre-existing shield against infection that provides protection during the lag period required for germinal center formation and adaptive antibody production in mice (43). IgG production profile remained similar in all experimental conditions (Figure 3A), indicating that although the B1 B-cell population is a minority, they are able to respond more rapidly and vigorously than conventional B2 B-cells in our experiments.

Our data showed that PRO-stimulated B1 B-cells from I patients present a balanced cytokine response, evidenced by the concomitant expression of TNF-α and IL-10 (Figures 2B–D). This balanced cytokine expression may reflect a self-regulated population which may also be contributing with the control of the immune response and the non-emergence of clinical symptoms in I patients. Several studies have shown the important role of cytokine-mediated immunoregulation in the maintenance of asymptomatic clinical forms during the progression of Chagas disease, mainly associated with the production of modulatory cytokines IL-10 and pro-inflammatory cytokines TNF-α and IFN-γ (22, 34, 44–47). While the asymptomatic (indeterminate) form represents a state of balance between modulatory and pro-inflammatory cytokines, the establishment of pathology represents the loss of this balance with consequent onset of tissue damage. The high frequency of TNFα^+^IL-10^+^ cells in I after PRO stimulation was an interesting finding that merits further analysis. Previous studies have suggested that the co-expression of inflammatory/anti-inflammatory cytokines by T cells may reflect a strategy of response to intracellular pathogens, providing and activated, self-regulating response (48).

In addition to the observed stimulation of B1 B-cells from indeterminate patients by PRO, we observed that these cells display a profile consistent with activation, as measured by the increase in the expression of CD11b (Figure 4B), a molecule of the integrin family that is a marker of B-cell activation, and important for B-mediated T-cell stimulation (10). It has been shown that CD11b participates in the maintenance of tolerance of auto-reactive B-cells in systemic lupus erythematosus (49), implying a protective role in autoimmune pathogenic conditions. In our study, CD11b expression by B1 B-cells was correlated with a better cardiac function of Chagas patients, based on LVDD and LVEF values. Decreased LVDD, as well as increased LVEF, have shown a correlation with the survival rate in Chagas patients (50, 51). Together, these data point to an association between the frequency of CD11b^+^ B1 B-cells and a favorable clinical outcome in Chagas disease (Figures 4D,E).

Given the suggested cross talk between CD11b^+^ B1 B-cells and TCR-gamma-delta T cells influencing their activation and function (29, 30), we evaluated the frequency of CD69^+^ (cell activation marker), TNF-α and IL-10 expression by CD4^+^ and CD8^+^ and double-negative (CD4^−^CD8^−^, DN) T cells expressing the TCR gamma-delta. We observed a higher frequency of CD4^+^ TCR gamma-delta^+^CD69^+^ cells in I patients both *ex vivo*, as well as after stimulation with PRO (Figures 5A,B). Gamma-delta T cells stimulated by B1 B-cells can produce cytokines and display cytotoxic functions (52–55), which can be protective or pathogenic, as shown in cancer and HIV-infection, respectively (56–58). Although in this study we did not evaluate the expression of molecules that may be associated with cytotoxic function of gamma-delta T cells, it is possible that the higher activation state of these cells found in I patients may be related with the control of *T. cruzi* via cytotoxicity. This would speak in favor of a protective role for CD4^+^gamma-delta^+^ T cells in helping to control parasitemia. Recent studies have shown that B1 B-cells can also induce differentiation of naive CD4^+^ T cells into IL-17–expressing T cells (59). Others and we have suggested a protective role for IL-17-expressing T cells in human Chagas disease (60, 61). Whether those protective Th17 cells express the gamma-delta TCR is yet to be determined. In addition, it is known that the vast majority of the peripheral TCR gamma-delta^+^ cells are DN. Previous studies by us have shown that these cells are associated with Chagas heart disease and that they are specifically activated by glycoconjugates (GCL fraction) from *T. cruzi* trypomastigotes (24). Thus, the fact that PRO did not increase the expression of CD69 by DN TCR gamma-delta T cells was not surprising. Interestingly, although PRO led to an increased expression of IFN-γ by cells from I, it also led to an increase in IL-10 by these cells from I, but not C nor NI, suggesting that PRO induces a balanced profile in DN T cells from I, which is consistent with their clinical outcome.

As opposed to the observed with CD4^+^gamma-delta^+^CD69^+^ T cells, we observed a lack of activation and even a decrease in frequency of CD8^+^gamma-delta^+^CD69^+^ T cells in I patients. On the other hand, the frequency of CD8^+^gamma-delta^+^CD69^+^ cells was higher in non-stimulated cultures of cells from C (Figure 5B). PRO did not significantly stimulate these cells. CD8^+^ T cells are the main cell type in the inflammatory infiltrate found in the heart of cardiac Chagas patients (62). It has been demonstrated that gamma-delta^+^ T cells can recognize autoantigens (63). The fact that CD8^+^gamma-delta^+^CD69^+^ T cells are increased in C patients, but are not expanded by the stimulus with PRO suggests that these cells may recognize either other parasite components or auto-antigens. If these cells are implicated in a pathogenic response, it is interesting that their frequency was lower in I patients. The mechanisms of activation and function of gamma-delta^+^ T cells is another topic that we intend on investigating further in future studies.

Unlike the findings of this study, which evidenced a protective role for B1 B-cells cells in chronic Chagas disease, previous studies in experimental models have demonstrated that CD5^+^ B-cells contribute to pathology (64, 65). More recently, it was shown that murine B1 B-cells can be classified into B1a and B1b according with the expression (CD5^+^) or lack (CD5^−^) of cell surface protein (66). Within this classification, it is proposed that B1a (CD5^+^) cells are able to recognize self-antigens and participate in autoimmune responses and, therefore, may be related to the observed pathogenic functions in experimental *T. cruzi* infection. It is possible that the use of fractionated antigens in the present study was responsible for the expansion of particular clones of B1 B-cells with protective characteristics. Thus, these findings may be useful in the development of immunotherapeutic strategies for the prevention/treatment of Chagas disease cardiomyopathy.

## Data Availability Statement

All relevant data for this study are contained within the manuscript.

## Author Contributions

LSAP conducted the experiments, analysis and data interpretation. LMDM collaborated in the execution of the experiments and parasite culture. RPS and AFM contributed with antigen fractionation efforts; MLRA and RCG contributed with the ELISAs. MCPN was the clinician responsible for the care, classification and enrollment of the patients. KJG and WD contributed to experimental design, analysis and data interpretation.

### Conflict of Interest Statement

The authors declare that the research was conducted in the absence of any commercial or financial relationships that could be construed as a potential conflict of interest.

## Abbreviations

BCR: B-cell receptor
BSA: bovine serum albumin
C: cardiac patients
Ch: chloroform
CD: Chagas disease patients
GCL: *T. cruzi*-derived glycolipid-enriched fraction
I: indeterminate patients
IL: interleukin
IFN: interferon
LIP: *T. cruzi*-derived lipid-enriched fraction
LVDD: left ventricular diastolic diameter
LVEF: left ventricular ejection fraction
M: methanol
NI: non-infected individuals
NS: non-stimulated
PBS: phosphate-buffered saline
PRO: *T. cruzi*-derived protein-enriched fraction
RPMI 1640: Roswell Park Memorial Institute medium 1640
TCR: T-cell receptor
TNF: tumor necrosis factor
W: water.
