# Corrigendum: Activation of Human CD11b^+^ B1 B-Cells by *Trypanosoma cruzi*-Derived Proteins Is Associated With Protective Immune Response in Human Chagas Disease

**DOI:** 10.3389/fimmu.2019.00367

**Published:** 2019-03-05

**Authors:** Livia Silva Araújo Passos, Luísa Mourão Dias Magalhães, Rodrigo Pinto Soares, Alexandre F. Marques, Marina Luiza Rodrigues Alves, Rodolfo Cordeiro Giunchetti, Maria do Carmo Pereira Nunes, Kenneth J. Gollob, Walderez Ornelas Dutra

**Affiliations:** ^1^Laboratory of Cell-Cell Interactions, Instituto de Ciências Biológicas, Departamento de Morfologia, Belo Horizonte, Brazil; ^2^Pós-graduação em Parasitologia, Universidade Federal de Minas Gerais, Belo Horizonte, Brazil; ^3^Laboratory of Cellular and Molecular Parasitology, Instituto René Rachou, Fundação Oswaldo Cruz, FIOCRUZ, Belo Horizonte, Brazil; ^4^Departamento de Clínica Médica, Faculdade de Medicina, Universidade Federal de Minas Gerais, Belo Horizonte, Brazil; ^5^Center for International Research, A.C.Camargo Cancer Center, São Paulo, Brazil; ^6^Instituto Nacional de Ciência e Tecnologia Doenças Tropicais, Belo Horizonte, Brazil

**Keywords:** B1 B-cells, Chagas disease, cardiomyopathy, *Trypanosoma-cruzi*, immunoregulation, cytokines

In the original article, we neglected to include the funder “National Institute of Allergy and Infectious Diseases of the National Institute of Health (NIAID-NIH), R01AI138230” to WD.

The authors apologize for this error and state that this does not change the scientific conclusions of the article in any way. The original article has been updated.

